# New Diagnosis of G6PD Deficiency Presenting as Severe Rhabdomyolysis

**DOI:** 10.7759/cureus.2387

**Published:** 2018-03-28

**Authors:** Akaolisa S Eziokwu, Dana Angelini

**Affiliations:** 1 Internal Medicine, Cleveland Clinic Ohio; 2 Hematology and Medical Oncology, Cleveland Clinic Ohio

**Keywords:** g6pd deficiency, rhabdomyolysis, hemolysis, exercise induced myoglobinuria, pigmenturia, myoglobinuria, glucose-6-phosphate dehydrogenase deficiency

## Abstract

A 24-year-old African-American man presented with malaise and low back pain and was found to have acute severe rhabdomyolysis followed by acute hemolysis. Glucose-6-phosphate dehydrogenase (G6PD) deficiency was suspected by the presence of blister cells on peripheral smear and was confirmed by a low enzyme activity assay. Our patient reported playing football, along with upper respiratory infection symptoms, prior to presentation. Extensive infectious and toxicology workup was negative; however, several inflammatory proteins were markedly elevated. We hypothesized the large inflammatory burden led to an increased reactive oxygen radical burden that overwhelmed muscle and erythrocyte reducing power. Severe rhabdomyolysis in G6PD deficiency is not a common presentation because skeletal muscles are more resistant to oxidative damage compared to red blood cells. Our case adds to the few existing reports of myolysis in the setting of G6PD deficiency.

## Introduction

Glucose-6-phosphate dehydrogenase (G6PD) deficiency is the most common enzyme deficiency worldwide and the best described red blood cell (RBC) enzymopathy [1]. It is an X-linked recessive disorder affecting about 400 million people globally with a high prevalence in Sub-Saharan Africa, Asia, Middle East, Latin America, and the Mediterranean [2]. In the United States, it is present in about 10% of African American males.

G6PD is expressed in all tissues where it catalyzes the first reaction in the pentose phosphate pathway which generates reduced nicotinamide adenine dinucleotide phosphate (NADPH). NADPH, in turn, produces reduced glutathione (GSH) which protects cells against oxidative stress [1]. Erythrocytes are particularly susceptible to oxidative damage in G6PD deficient individuals because, unlike other tissues, they lack mitochondria and therefore depend solely on G6PD to generate NADPH. 

Many G6PD deficient persons are asymptomatic. The disease commonly manifests as neonatal hyperbilirubinemia within first 24 hours of life or acute hemolysis induced by oxidative stress. Common triggers include ingestion of fava beans, infections, or exposure to certain drugs. Less commonly, G6PD deficiency can manifest as a chronic non-spherocytic hemolysis [1]. Muscle manifestations from a G6PD deficiency are not a well-recognized complication and only a few cases have been reported.

We describe a 24-year-old male who was found to have G6PD deficiency during an evaluation of acute severe rhabdomyolysis and hemolytic anemia. Reports of similar cases are reviewed in the discussion.  

## Case presentation

A 24-year-old African-American male with no significant past medical history was admitted to our hospital as a transfer from another facility for the further management of rhabdomyolysis. He first presented to the outside hospital with a two-day history of malaise, mild exertional shortness of breath, low backache, and subjective fevers. He endorsed upper respiratory symptoms and also reported playing football with friends several days prior to presentation. He denied a history of trauma, strenuous activity, sick contacts, recent travel, or ingestion of any medication or fava beans. He endorsed using marijuana but denied cigarette smoking, alcohol, or other recreational drugs. Review of systems was otherwise negative. He had no known drug allergies and no significant family history.

Physical examination revealed a blood pressure of 154/66 mmHg, heart rate of 105/minute, a temperature of 98.3°F (36.8°C), a respiratory rate of 18/min, and an oxygen saturation of 99% on room air. The oral mucous membrane was dry, and there was no scleral icterus. Lung and abdominal examination were unremarkable, and he was alert and fully oriented.

Laboratory results upon admission to the initial hospital were notable for the following: hemoglobin (Hb) 13.6 g/dL (12.5 - 16.5), white blood cell (WBC) count 2.7 x 10⁹/L (4.5 - 11.5) (differential notable for lymphocyte count 0.11 x 10⁹/L (1.5 - 4.0)), platelet count 103 x 10⁹/L (130 - 450), prothrombin time (PT) 14.8 seconds (9.3 - 12.4), activated partial thromboplastin time (aPTT) 28.1 seconds (24.5 - 35.1), peripheral smear reported normocytic anemia, lymphopenia, rare schistocytes, and normal platelet number and morphology; C-reactive protein (CRP) 5.2 mg/dL (0.0 - 0.4),  creatinine 1.3 mg/dL (0.7 - 1.2), blood urea nitrogen (BUN) 14 mg/dL (6 - 20), bicarbonate 21 mmol/L (22 - 29), total bilirubin 4.8 mg/dL (0.0 - 1.2), direct bilirubin 2.1 mg/dL (0.0 - 0.3), indirect bilirubin 2.7 mg/dL (0 - 1.0), aspartate amino transaminase (AST) 247 U/L (0 - 39), alanine aminotransferase (ALT) 192 U/L (0 - 40), urine myoglobin 782 mg/L (0 - 1), total creatine kinase (CK) 9,916 U/L (20 - 200), aldolase 25.5 U/L (1.5 - 8.1), thyroid-stimulating hormone (TSH) 1.37 IU/mL (0.27 - 4.2), glucose 101 mg/dL (74 - 109), and urine toxicology screen only positive for cannabinoids. Respiratory virus film array was negative for influenza A and B, coronavirus, adenovirus, metapneumovirus, parainfluenza virus, respiratory syncythial virus (RSV), rhinovirus, Bordetella pertussis, Chlamydophila pneumoniae, and Mycoplasma pneumoniae. Hepatitis viruses, human immunodeficiency virus 1 and 2, and urine and blood cultures were also negative.

Right upper quadrant (RUQ) ultrasound (US) showed no hepatic abnormality, a renal US was negative for hydronephrosis, and computed tomography (CT) abdomen and pelvis without contrast was negative for any acute pathologies. He received intravenous rehydration, empiric oseltamivir (pending viral workup), and empiric antibiotics.

Over the course of his five-day stay at the outside facility, his clinical course worsened and his hemoglobin had progressively declined to 8.7 g/dL, while his WBC count and platelet count had normalized. CK peaked at 373,240 U/L, creatinine rose to 2.3 mg/dl, BUN at 31 mg/dL, total bilirubin at 18 mg/dL, and direct bilirubin at 16 mg/dL. AST and ALT values were 3,288 U/L and 754 U/L, respectively. He also developed progressively worsening shortness of breath requiring oxygen supplementation with nasal cannula. On account of his deteriorating clinical course, he was transferred to our hospital.

Initial workup at our institution revealed a hemoglobin of 7.7 g/dL, WBC count 9.17 x 10⁹/L, platelet count 164 x 10⁹/L, reticulocyte 2.2% (0.2 - 2.0) with a peak value of 14.5% five days later, CK 315,441 U/L, total bilirubin 21.3 mg/dL, direct and indirect bilirubin of 15.5 mg/dL and 5.8 mg/dL, respectively, AST 2,737 U/L, ALT 704 U/L, creatinine 2.447 mg/dL, BUN 32 mg/dL, LDH 7,650 U/L, haptoglobin < 10%, serum albumin 2.3 mg/dL, ferritin 24,718 ng/mL (30.3 – 565.7), CRP 11.8 mg/dL (< 0.9), and anti-neutrophil antibody (ANA) negative by enzyme immunoassay (EIA). Direct Coombs test was negative. Peripheral smear was significant for a moderate number of blister cells. G6PD quantitative assay was 3.8 U/g Hb (8.6 - 18.0) with normal hemoglobin electrophoresis.

He had intermittent fevers during his stay, and an extensive bacterial and viral infectious workup was obtained which was unremarkable. He received supportive care with intravenous fluids and eventual discontinuation of empiric antibiotics. His clinical course and laboratory values slowly improved and he was discharged home 10 days later. He was followed up in hematology clinic 11 days after discharge and repeat labs showed significant improvements in the anemia and acute kidney injury with a hemoglobin of 12.2 g/dL and creatinine of 1.29 mg/dL, respectively; other laboratory improvements were reticulocyte 1.1%, total bilirubin 2.7 mg/dL, ALT 123 U/L, AST 57 U/L, and CK 137 U/L. Repeat G6PD assay remained low at 3.5 U/g Hb.

## Discussion

Our patient presented with severe rhabdomyolysis followed by acute hemolytic anemia and renal failure. A peripheral blood smear showed blister cells and a G6PD activity assay confirmed the G6PD deficiency. The precipitant of his rhabdomyolysis remained unclear at the time of discharge, given the negative infectious and toxicology workup. Nevertheless, his very high serum ferritin, CRP, and low albumin were reflective of a very inflamed internal milieu, which generated significantly large quantities of oxidative radicals that overwhelmed muscle and erythrocyte protective reducing power. He reported playing football with friends, along with a viral prodrome, a few days prior to his presentation so physical exertion, in addition to an unidentified oxidative stressor(s), potentially triggered his symptomatology. Other potential causes of rhabdomyolysis, such as inflammatory myopathies, endocrinopathies (hypothyroidism or diabetes mellitus), neuroleptic malignant syndrome, and seizures, were unlikely in our patient on the basis of clinical and laboratory data. Metabolic myopathies due to inborn errors of glycogen or lipid metabolism can rarely present as rhabdomyolysis but more commonly manifest as episodes of exercise-induced muscle pain and/or exercise intolerance which was not present in our patient.

Acute hemolysis from G6PD deficiency usually manifests 24 - 72 hours after exposure to oxidant stress. It is plausible the metabolic acidosis induced by acute renal failure, acting in combination with an underlying oxidative trigger, precipitated his acute hemolysis. It is important to note G6PD levels can be falsely normal during an acute hemolytic episode because reticulocytes have higher G6PD activity than mature red blood cells, which necessitates repeat testing after the resolution of hemolysis if there is high clinical suspicion of G6PD deficiency. Interestingly, our patient had low levels of G6PD during the hemolytic crisis which remained low after resolution of hemolysis, strongly suggesting a true G6PD deficiency. The etiology of his absolute lymphopenia remained unclear but was likely related to the undiagnosed underlying infectious/inflammatory trigger; conversely, the mild thrombocytopenia seen on presentation was likely a false positive result, given that platelets were normal on follow-up peripheral smear. Rhabdomyolysis can be expected to cause elevation of ALT and AST; however, the presence of significant conjugated hyperbilirubinemia in our patient was suggestive of some component of acute hepatocellular injury of unclear etiology contributing to his liver enzyme elevation.

G6PD levels in skeletal muscle cells have been shown to correlate with enzyme activity in erythrocytes [3]. However, skeletal muscles are much more resistant to oxidative damage than RBCs of G6PD-deficient persons because muscle cells possess alternate enzymes, such as catalase and superoxide dismutase, which are absent in erythrocytes. Nevertheless, skeletal muscles have lower levels of catalase and superoxide dismutase compared to other tissues and have some dependency on glutathione to remove reactive oxidative radicals [3]. In a mouse model, muscle glutathione needed to be severely depleted to induce skeletal muscle breakdown [4]. This finding suggests that the burden of oxidative stress needs to be very large in order to overwhelm glutathione and alternative pathways and cause muscle damage. This probably explains the rarity of severe muscle manifestations in G6PD-deficient patients and why there were no significant differences in two studies that compared blood and muscle oxidative stress markers between G6PD-deficient and normal individuals after they had been subjected to moderate [5] or severe [6] physical exertion.

Bresolin et al. reported a case of a 30-year-old pentathlon-trained athlete with a previous history of exercise-induced pigmenturia, who was diagnosed with a G6PD deficiency when he presented with unconsciousness, rhabdomyolysis, and hemolysis after running about 12 Km [7]. The same authors also published three other cases of males who were diagnosed with a G6PD deficiency during evaluation of recurrent unexplained myalgias, muscle fatiguability, or exercise-induced myoglobinuria [7]. Two other cases of myolysis and hemolysis in African-American (AA) male patients with sickle cell trait (SCT) and G6PD deficiency were also reported. One was a 34-year-old Black male diagnosed with sickle cell trait and G6PD deficiency after presenting with rhabdomyolysis and acute hemolytic anemia 24 hours after vigorous exercise [8]. The other case was a two-year-old AA male with SCT diagnosed with a G6PD deficiency after he developed rhabdomyolysis and acute hemolysis on exposure to a mothball [9]. In these two cases, G6PD deficiency in concert with SCT likely precipitated rhabdomyolysis and hemolysis. G6PD deficiency can occur simultaneously with SCT in up to 5% of men of African descent [10] and thus should be investigated when male patients of African or African-American descent with SCT present with unexplained severe rhabdomyolysis and/or hemolysis.

## Conclusions

In conclusion, we report a 24-year-old African-American male patient who presented with rhabdomyolysis and acute hemolytic crisis found to have a G6PD deficiency. No clear trigger for his presentation was identified after an exhaustive workup but a viral prodrome and physical activity were notable findings in his case. Severe manifestations of muscle damage in G6PD deficiency is rare in medical literature, and our case adds to the limited reports available. We encourage clinicians to consider a G6PD deficiency in the differential diagnosis of unexplained rhabdomyolysis and/or hemolysis in male patients of African, African-American, Asian, or Mediterranean descent.
